# Procalcitonin levels in children affected by severe malaria compared to those with uncomplicated malaria in the absence of bacterial infection: a cross-sectional study

**DOI:** 10.1186/s40794-022-00163-9

**Published:** 2022-03-15

**Authors:** Jean-Claude Katte, Kiya Penanje, Batakeh B. Agoons, Eric Noel Djahmeni, Sharon Mbacham-Ngwafor, Vicky Jocelyne Ama Moor, Paul Koki, Wilfred Mbacham

**Affiliations:** 1 Department of Public Health, Faculty of Medicine and Biomedical Sciences, University of Yaounde 1, Yaounde, Cameroon; 2 National Obesity Centre and Endocrinology and Metabolic Diseases Unit, Yaounde Central Hospital, Yaounde, Cameroon; 3 Department of Biochemistry, Faculty of Medicine and Biomedical Sciences, University of Yaounde 1, Yaounde, Cameroon; 4 Mother and Child Centre, Chantal Biya Foundation, Yaounde, Cameroon; 5 Laboratory for Public Health Biotechnologies, the Biotechnology Centre, University of Yaounde 1, Yaounde, Cameroon

**Keywords:** Bacterial infections, Child, Severe malaria, Procalcitonin

## Abstract

**Background:**

Procalcitonin is an inflammatory marker strongly associated with the presence of bacterial infection. It has been considered raised in severe malaria infection as opposed to uncomplicated malaria. There are suggestions that it may be raised only when there is concomitant unnoticeable bacterial infection during a malaria crisis. We aimed to assess the difference in plasma procalcitonin levels between children affected by severe and uncomplicated malaria.

**Methods:**

We assessed plasma procalcitonin levels in 83 children diagnosed with malaria with no clinical and biological evidence of concomitant bacterial infection. Severity of malaria was established using WHO guidelines. Procalcitonin was determined using the ELISA method. Non-parametric Mann-Whitney U test was used to compare medians across the 2 groups. Statistical significance was set for all *p* values < 0.05.

**Results:**

Of the 83 participants, 28 had uncomplicated malaria, and 55 had severe malaria. PCT levels were obtained in 24 and 40 subjects of each group, respectively, and were similar in both groups; [2.76 (2.52–2.93) vs 2.74 (2.52–2.98) ng/ml, *p* = 0.916]. The parasite density was lower in the uncomplicated malaria group than in the severe malaria group, but not statistically significant; [22,192 (9110–44 654) vs 31 684 (13 960–73 500) parasites/μl, *p* = 0.178]. There was no correlation between the parasite density in the general study population and PCT levels (r = 0.072, *p* = 0.572).

**Conclusion:**

In the absence of overt bacterial infection, procalcitonin levels are not different between children affected with uncomplicated malaria and those with severe malaria. Therefore, bacterial infection should be thoroughly checked for in children with raised serum procalcitonin diagnosed with severe malaria.

## Introduction

Procalcitonin (PCT) is currently known to be the standout marker for the diagnosis of bacterial infections as higher levels of serum PCT are found in patients with severe bacterial infections in comparison with patients with viral infections and other inflammatory conditions relative to viral infections and nonspecific inflammatory diseases [1]. PCT is a 116 amino acid precursor of the hormone, calcitonin. It is produced in para-follicular cells (C cells) of the thyroid and by the neuroendocrine cells of the lung and intestines [2]. Some studies also have shown it to be raised in fungal infections and parasitic infections such as malaria [3]. Plasmodium falciparum malaria is endemic in Africa and has a great mortality outcome in the pediatric group [4]. Indeed, Children aged under 5 years accounted for 67% (274000) of all malaria deaths worldwide, in 2019 [5]. As its clinical course mimics some bacterial infections, early diagnosis and recognition of its severity status is an important challenge for healthcare professionals in this geographical area. Malaria can generally be classified clinically into uncomplicated malaria (UM) and severe malaria (SM) depending on the World Health Organization (WHO) severity criteria [6]. However, accurate interpretation of these criteria is dependent on subjective assessment. As a result of malaria infection involving dysregulated inflammatory responses, it is hypothesized that plasma inflammatory biomarkers might have clinical importance as an assessment tool [7]. Therefore, there is a growing interest in the need for reliable discriminatory markers in children.

Serum PCT has previously been shown to be raised in malaria infections and serves as a good discriminatory tool between uncomplicated malaria and severe malaria in the adult population [8–10]. Whether this is true in a paediatric population is not very certain since very few studies have been conducted in this specific age group and with conflicting results [11–13]. We therefore aimed to examine whether there exists any differences in serum PCT levels between children infected and diagnosed with uncomplicated malaria compared to those with severe malaria in Cameroon.

## Methods

### Study design, setting and participants

**Fig. 1:**
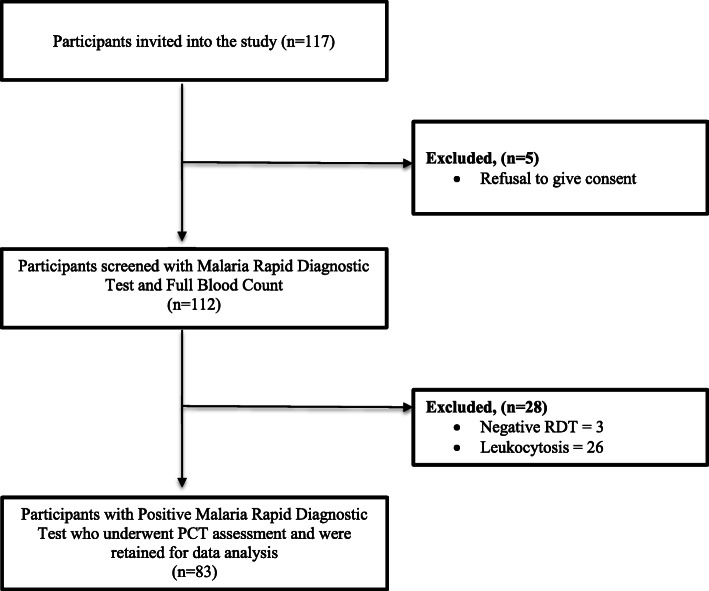
Flow diagram of participant recruitment in the study

The study was a cross-sectional, descriptive, and analytical study, carried out at the Mother and Child Centre (MCC), Chantal Biya Foundation, Yaoundé, Cameroon, over a period of 7 months (November 2018 to June 2019). A total of 126 children (aged 6 months to 15 years) were initially selected based on presenting complaints. The children to take part in the study were selected by a single paediatrician who thoroughly examined the children for a concomitant clinical focus of bacterial infection. Those with overt evidence of clinical bacterial infections were excluded. These included anomalies to various organ systems such as bulging tympanic membranes, hyperemic conjunctiva, erythematous oropharynx, lung crackles, lymphadenopathy and present costovertebral and supra-pubic tenderness. A complete blood count was also performed to exclude all cases with hyper leukocytosis. A total of 83 children were retained for the study. Figure 1 shows the flow diagram of how the study participants were enrolled into the different phases of the study.

### Assessment of malaria using a rapid diagnostic test for malaria

This was done using rapid diagnostic test (RDT) kits for malaria (*Standard Diagnostics BIOLINE®, Malaria Ag. P.f/Pan)* with peripheral venous blood which had been collected into a 3-5 ml EDTA tube. Whole blood (0.5 mL) was pipetted into the EDTA tube and dropped in the sample hole quickly enough to avoid blood clotting. Two drops of the buffer were put into the appropriate hole. After waiting for 15–20 minutes, the results were read. The RDT was considered valid with the presence of the control line.

### Peripheral blood smear for malaria and assessment of severity

Parasite density was evaluated by May-Grunwald and Giemsa stains of thin and thick smears, respectively, using blood from the EDTA tubes. The parasite density was calculated per microlitre of blood using each patient’s white blood cell count from a full blood count analysis. (*©2017 Shenzhen Mindray BC 3D Bio-medical Electronics Co. Ltd)*. Thin films were examined only for malaria parasite species. Severe malaria was defined using the WHO malaria severity criteria [6]: impaired consciousness, prostration, convulsions, acidosis, hypoglycaemia, severe anemia, renal impairment, jaundice, pulmonary edema, significant bleeding, shock, hyperthemia at 40^o^ c and hyperparasitemia. Severe malaria was diagnosed if one or more criteria for severity were present.

### Assessment of plasma procalcitonin levels

Blood collected in the EDTA tubes was centrifuged, plasma was obtained and stored in a − 80 °C freezer in cryotubes pending batch analysis. Analysis was completed within 2 months of storage. The samples did not undergo any freeze-thaw process before analysis. Plasma PCT was then measured using Human PCT Enzyme-Linked Immunosorbent Assay (ELISA) Kit. Catalog No: E-EL-H1492 96 Test Elabscience® following the manufacturer’s instructions at the Clinical Diagnosis Laboratory of the National Obesity Centre, Yaoundé Central Hospital. The sensitivity of the assay was 18.75 pg/ml. The kit was able to recognise natural and some recombinant human PCT. No significant cross-reactivity or interference between human PCT and analogues was observed. The coefficient of variation (precision within an assay and between assays) was < 10%.

### Study outcome variables

The primary outcome was the difference in serum PCT levels between the cases with uncomplicated and severe malaria. Other secondary outcome variables included presenting complaints at inclusion into the study and malaria parasite density.

### Statistical analysis

Data was entered with Epi info version 7.2.2.6 *and* analysed with SPSS (Statistical Package for Social Sciences) version 22.0. Student t-test was used to compare means while Mann-Whitney U test was used to compare medians. Correlation between continuous variables was examined using Spearman correlation coefficient. *P* values less than 0.05 were considered statistically significant.

### Ethical consideration and confidentiality

Ethical clearance was obtained from the Institutional Review Board (IRB) of the Faculty of Medicine and Biomedical Sciences (FMBS) – University of Yaoundé I (N^o^: 117/UY1/FMSB/VDRC/CSD), Centre Regional Ethics Committee for Human Research (N^o^: 1582/AP/MINSANTE/SG/DRSPC/CRERSH) and the parents/guardians of the participants provided a signed written informed consent before participating in the study.

## Results

### Baseline characteristics of the study population

Table 1 shows the socio-demographic characteristics of the study cases. Of the 83 participants finally recruited into the study, 45 were males. Children aged 5 years and below were the most represented age group (77.1%). The majority of the cases (95.2%) had received some form of treatment before seeking for healthcare, while 33.7% of them had taken an anti-malarial agent prior to consultation. The most common anti-malarial agent used was Oral Artemisinin-based combination therapy (ACT) in half (53.6%) of the cases.

**Table 1:** Table showing the socio-demographic characteristics of the study population

Participant characteristics	Frequency	Proportion (%)
Gender		
Male	45	54.2
Female	38	45.8
Age group		
Less than 1 year	42	50.6
1–5 years	22	26.5
6–10 years	10	12.0
Greater than 10 years	9	10.8
Place of residence		
Urban	67	80.7
Rural	16	19.3
Level of education of parent		
None	1	1.2
Primary	13	15.7
Secondary	51	61.4
University	18	21.7
Use of mosquito net at home		
Yes	63	75.9
No	20	24.1
Is this the first malaria episode?		
Yes	21	25.3
No	62	74.7
Number of prior malaria episode		
Less than 5	52	62.7
5–10	10	12.0
Any prior anti-malarial treatment received before consultation		
Yes	28	33.7
No	55	66.3
Type of antimalarial taken		
Oral ACT	15	53.6
Oral quinine	3	10.7
Injectable quinine	5	17.9
Arthemeter/Artesunate	5	17.9

*ACT* Artemisinin-based combination therapy

### Presenting complaints at enrolment into the study

**Fig. 2:**
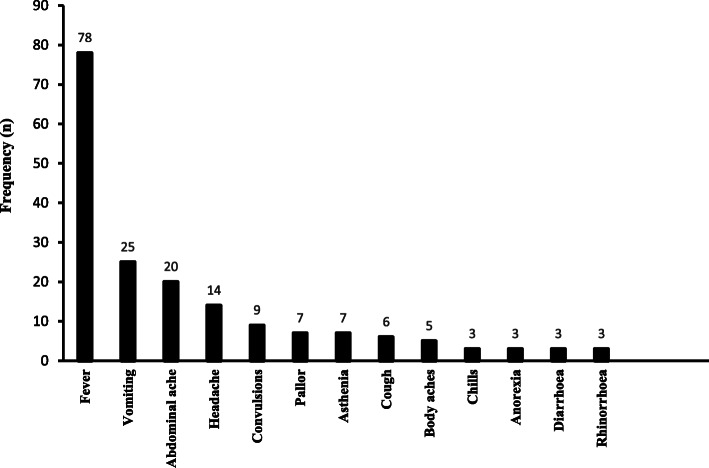
Presenting complaints at enrolment into the study

Figure 2 shows the presenting complaints at enrolment into the study. The majority of the cases 78 (94.0%) presented with fever. The frequency of other common presenting complaints was 25 for vomiting (30.1%), 20 for abdominal ache (24.1%), 09 for headaches (16.9%), 09 for convulsions (10.8%), and 07 for pallor (8.4%).

### Diagnosis and severity of malaria

All cases enrolled into the study had a positive RDT result for malaria and showed a positive smear for *P. falciparum*. Based on the WHO clinical severity criteria for malaria, 28 (33.7%) and 55 (66.3%) participants were diagnosed with uncomplicated and severe malaria, respectively, as seen in Table 2. The median (IQR) parasite density in the study population was 26,800 (12200–56,700) parasites/μl. Those with uncomplicated malaria had a similar parasite density 22,192 (8932–45,048) parasites/μl compared to the cases with severe malaria, 31,684 (13960–73,500) parasites/μl, *p* = 0.178. The platelet counts were lower in participants with severe malaria compared to those with uncomplicated malaria [108 (66–152) vs 172.5(102.8–275.3) *p* = 0.004]. All other full blood count parameters (white blood cell counts, haemoglobin levels) showed no significant changes between groups.

**Table 2:** Biological characteristics of the study participants stratified by severity of malaria

Characteristics	Uncomplicated Malaria (***n*** = 28)	Severe Malaria (***n*** = 55)	***P*** value
WBC count, median (IQR), cell/mm^3^	9400 (7400–11 000)	9850 (7900–10 800)	0.578
Haemoglobin level, median (IQR), g/dL	9.5 (8.0–11.2	9.5 (7.2–11.0)	0.287
Platelet count, median (IQR), cell/mm^3^	172.5 (102.8–275.3)	108 (66–152)	0.004
*P. falciparum* parasite density, median (IQR), parasite/μl	22 192 (9110–44 654))	31 684 (13 960–7 3500)	0.178
Procalcitonin level, median (IQR), ng/mL	2.76 (2.52–2.93)	2.74 (2.52–2.98)	0.916

### Difference in PCT levels by malaria severity

Serum PCT values were finally obtained in 64 patients; 24 and 40 cases with uncomplicated and severe malaria. Nineteen (19) cases had undetectable serum PCT levels (below the limit of detection of the kit used) Among those with undetectable serum PCT levels, 10 had severe malaria and 9 had uncomplicated malaria. The median (IQR) serum PCT level in the study was 2.75 (2.52–2.96) ng/ml. The median (IQR) serum PCT level in the cases with uncomplicated malaria was 2.76 (2.52–2.93) ng/ml while in those with severe malaria was 2.74 (2.52–2.98) ng/ml. There was no statistically significant difference in the serum PCT levels between both malaria groups (*p* = 0.916) as shown in Table 2. There was no correlation between the parasite density in the general study population and PCT levels (r = 0.07, *p* = 0.57). In the uncomplicated malaria group, there was no meaningful correlation (r = − 0.03, *p* = 0.89) between the parasite density and serum PCT values (data not shown).

Table 3 shows the characteristics of the study participant stratified by those who received prior antimalarial treatment in the group with uncomplicated malaria and severe malaria. Among the cases with uncomplicated malaria, there was no difference in the serum PCT levels between those who had received prior antimalarial treatment and those who did not; 2.48 (2.17–2.88) ng/mL vs 2.82 (2.55–2.93) ng/mL, *p* = 0.43. Also the white blood cell count, platelet count, haemoglobin level and parasite density were similar between the two groups. Also, among the cases with severe malaria, the serum PCT levels was similar between those had received prior antimalarial treatment and those who had not; 2.65 (2.45–2.94) ng/mL vs 2.82 (2.55–2.97) ng/mL, *p* = 0.53. However, the parasite density was significantly lower among those with severe malaria who had received prior antimalarial treatment compared to those who had not; 18 869 (5025–32 737) parasite/μl vs 49 989 (22 641–108 475) parasite/μl, *p* = 0.005. The white blood cell count, plate count and haemoglobin level did not show any significant changes.

**Table 3:** Biological characteristics of the study participants stratified by severity of malaria and those who received prior antimalarial treatment

	Uncomplicated Malaria (***n*** = 28)		***P*** value	Severe Malaria (***n*** = 55)		***P*** value
	No Treatment (***n*** = 24)	Prior Treatment (***n*** = 4)		No Treatment (***n*** = 32)	Prior Treatment (***n*** = 23)	
**WBC count, median (IQR), cell/mm**^**3**^	7000 (5700–8100)	5900 (4900–9500)	0.97	7300 (5900–8600)	6700 (4600–9100)	0.64
**Hemoglobin level, median (IQR), g/dL**	9.4 (8.0–11.5)	9.7 (8.2–10.0)	0.83	9.4 (7.0–11.0)	10.1 (7.7–10.8)	1.00
**Platelet count, median (IQR), cell/mm**^**3**^	178 (114–278)	167 (67–267)	0.65	100 (70–142)	111 (62–193)	0.93
***P. falciparum*** **parasite density, median (IQR), parasite/**μl	22 173 (9466–45 443)	23 400 (5700–31 966)	0.90	49 989 (22 641–108 475)	18 869 (5025–32 737)	0.005
**Procalcitonin level, median (IQR), ng/mL**	2.82 (2.55–2.93)	2.48 (2.17–2.88)	0.43	2.82 (2.55–2.97)	2.65 (2.45–2.94)	0.53

## Discussion

We aimed to assess the difference in serum PCT levels in children aged between 6 months to 15 years diagnosed with uncomplicated and severe malaria in a clinical setting in sub-Saharan Africa. We found that 33.7 and 66.3% of our participants had uncomplicated and severe malaria, respectively, and that there was no significant difference in the serum PCT levels between these 2 groups. Serum PCT levels are usually very high in bacterial infections and therefore it can be used to confidently discriminate between bacterial infections and severe malaria given that high serum PCT in severe malaria cases is not supported by this finding, contrary to other studies.

Most participants in our study presented with fever at consultation as was reported by Trampuz et al. to be the most common presenting symptom in about 92% of malaria cases [14]. The majority of patients in our study were those with severe malaria. This was also the case in studies carried out by Mohapatra et al and Erdman et al [8, 12]. This can be explained by the fact that, sometimes severe malaria progresses so rapidly in children that treating uncomplicated malaria is not feasible and the diagnosis is made when patients already present with signs of severity [15]. This finding is similar to the WHO malaria report in 2018 which registered *P. falciparum* in all malaria cases recorded in Cameroon during the year 2017 [16]. We observed that the median parasite density in our study population was lower than that observed by Hollenstein et al. who had a higher median parasite density in his study population of 290 680 (533–1 147 040) parasite/μl [17]. The median parasite densities in the severe and uncomplicated malaria patients in our study were both lower than those found by Erdman et al [12]. The onset of malaria treatment in about one-third of our patient population prior to consultation at the hospital could also be a reason for the lower parasite density in our study population, compared to others [13, 17].

The median serum PCT value in our study population was higher than the value in a normal subject (< 0.2 ng/ml) as seen in most studies done earlier [8, 10, 17–19]. This elevation could be in response to an inflammatory process triggered in response to acute malaria infection [11, 17]. Raised values were considered above 0.2 ng/ml because 3 days following birth, serum PCT values in neonates and children are similar to those in adults [20]. Hyper-parasitemia (defined as more than 5% or 250,000 /μl in areas of high stable malaria transmission intensity) is a laboratory criterion for severe malaria and has been shown to be a good reflector of malaria outcomes. Serum PCT levels did not correlate with parasite density in this study. This is different from what was observed in studies carried out by Hesselink et al and Hollenstein et al. in that serum PCT values correlated with parasite densities [17, 21]. The median serum PCT level in the severe malaria group was not different compared to the uncomplicated malaria group in our study, contrary to the results obtained from studies carried out by Mohapatra et al, Chiwakata et al*,* Righi et al*,* Te Witt et al and Hesselink et al [8, 10, 18, 21, 22]. Comparing this to Erdman et al. who did a study in a Ugandan paediatric population, they had a significant difference in serum PCT levels between severe and uncomplicated malaria patients unlike ours. The higher levels in these other studies which could account for the correlation compared to our study, could be explained by the fact that most patients had their serum PCT levels analysed without haven taken any prior antimalarial treatment. In agreement, Braun et al. observed something similar to that in our study in a Ghanaian paediatric population in which nearly half (42%) of the symptomatic children in their study population took chloroquine before consultation at the hospital [11]. This could explain the similarity in their study and ours. In this study, in order to avoid prior usage of anti-malarial drug being a potential confounder, subgroup analyses stratifying according to prior anti-malarial usage was performed, and revealed no significant results.

Our study has some limitations. The small sample size of the study could have affected the ability to show significant differences between subjects with uncomplicated and severe malaria. In addition, PCT serum levels have been shown to possess rapid kinetics, with an early rise and fall in serum levels during a given infection [23, 24]. This study did not account for malaria disease duration when measuring PCT levels. Also, the usage of a single paediatrician to rule out clinical signs of infection may have  included some selection bias.  However, internal validity of this study was preserved as the paediatrician was an independent examiner and was unaware of the study outcomes. To exclude concomitant bacterial infections, this study used full blood count leucocytosis instead of the more specific blood cultures [25] due to cost constrains.

Nevertheless, the findings presented in this study are of clinical interest to health practitioners in this region of the study; in the absence of overt bacterial infection, serum PCT levels are not different between patients diagnosed with uncomplicated malaria and those with severe malaria. This finding may therefore inform the practice of diligently seeking to prove an associated bacterial infection in patients diagnosed with uncomplicated/severe malaria who have high PCT levels as a high PCT level may not be as a result of the severity of the malaria infection. Further larger sample size studies including children with confirmed bacterial infections and those with confirmed malaria infections or both are needed to better inform on the association between procalcitonin and malaria infections.

## Conclusion

In the absence of overt bacterial infection, serum procalcitonin levels are not different between children affected with uncomplicated malaria and those with severe malaria. Therefore, bacterial infection should be thoroughly checked for in children with raised serum procalcitonin diagnosed with severe malaria.

## Data Availability

The datasets generated and/or analyzed during the current study are not publicly available (due to the absence of an adequate privacy-ensuring public repository in the country). However, they are available from the corresponding author on reasonable request.
